# Surface Probe Linker with Tandem Anti-Fouling Properties for Application in Biosensor Technology

**DOI:** 10.3390/bios10030020

**Published:** 2020-03-03

**Authors:** Sandro Spagnolo, Brian De La Franier, Tibor Hianik, Michael Thompson

**Affiliations:** 1 Faculty of Mathematics, Physics and Informatics, Comenius University, Mlynská dolina F1, 842 48 Bratislava, Slovakia; sandrospagnolo1@gmail.com (S.S.); tibor.hianik@fmph.uniba.sk (T.H.); 2 Department of Chemistry, University of Toronto, 80 St. George Street, Toronto, ON M5S, Canada; brian.delafranier@mail.utoronto.ca

**Keywords:** anti-fouling monolayer, electromagnetic piezoelectric acoustic sensor, blood serum, milk

## Abstract

This paper describes the anti-fouling capability of the novel monolayer-forming surface linker 3-(3-(trichlorosilylpropyloxy) propanoyl chloride (MEG-Cl). This compound was successfully attached to quartz crystal surfaces which are employed in an electromagnetic piezoelectric acoustic sensor (EMPAS) configuration. The MEG-Cl coated surface was both employed with Ni-NTA for the binding of recombinant proteins and for the tandem property of the avoidance of fouling from serum and milk. The MEG-Cl coated surfaces were found to provide a large degree of anti-fouling on the EMPAS device, and were comparable to previously studied MEG-OH surfaces. Importantly, the monolayer continued to provide anti-fouling capability to the biosensor following extension with Ni-NTA in place. Accordingly, this surface linker provides an attractive system for use in biosensor technology in terms of both its anti-fouling and linking properties.

## 1. Introduction

Biosensors of whatever transduction type are well-recognized to suffer severely from interference with respect to both response selectivity and quantitative signaling when placed in biological fluids such as blood, plasma, serum, urine or cerebrospinal fluid [1]. This is caused, in part, by the non-specific adsorption (NSA) or fouling of the device substrate surface by proteinaceous species and cells present in such fluids. This process can both seriously hinder the performance of a biosensor by the instigation of a large background signal or through exclusion of interaction of the target analyte with the device-bound probe such as an antibody or nucleic acid [1]. With respect to various applications, including biosensor technology, numerous attempts have been made to prevent or at least reduce adsorption of biological entities to surfaces including biological macromolecules and cells. These efforts involving various classes of surface coatings or modifications such as the use of amino acids, peptides and peptoids; poly(ethylene glycol)-based coatings; zwitterionic self-assembled monolayers (SAMs); and carbohydrate derivatives have been the subject of several reviews [2,3,4].

**Figure 1:**
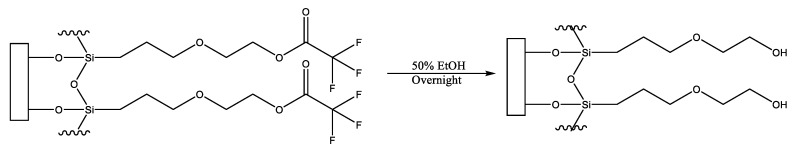
Conversion of surface bound MEG-TFA to MEG-OH.

An interfacial chemistry that can be modified to provide a surface possessing anti-fouling properties is that produced via the covalent attachment of the precursor molecule, 2-(3-trichlorosilylpropyloxy)-ethyl trifluoroacetate [5,6]. This molecule readily forms a monolayer on hydroxylated surfaces which can be hydrolyzed with facility to generate a distal hydroxyl moiety (on a surface, termed MEG-OH) (Figure 1). It has been found to provide favourable anti-fouling to surfaces compared to other tested surface layers [3,5].

The important anti-fouling capability of MEG-OH is clearly associated with the presence of the mid-chain ether group that is considered to enable the formation of an interfacial layer of hydration. Study of MEG-OH by neuron reflectrometry indicates that a relatively thick transition zone of water exists continuously with the ultrathin (<1 nm) adlayer prepared on Si [7]. Conversely, this physically distinct phase is thinner and only interfacial in nature for the adlayer lacking internal ether oxygen atoms. Molecular dynamic calculations of the MEG-OH system strongly suggest that the interfacial water layer exhibits reduced lability [8]. In normal biosensor operation, the MEG-OH structure is not employed for probe attachment, but is incorporated into a mixed monolayer ensemble with a linker for such attachment thus combining anti-fouling chemistry with selectivity towards an analyte.

**Figure 2:**
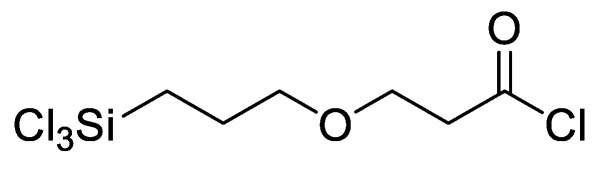
3-(3-(trichlorosilylpropyloxy)propanoyl chloride (MEG-Cl).

In our previous research, a functionalizable surface linker, 3-(3-trichlorosilylpropyloxy) propanoyl chloride (MEG-Cl), was developed and characterized (Figure 2) [9]. This linker was found to bind readily to hydroxylated glass surfaces, and could be extended with *N*_α_,*N*_α_-bis(carboxymethyl)-l-lysine (ab-NTA) to reversibly bind a recombinant protein to a glass surface. This linker was designed to be structurally similar to MEG-OH, with a similar chain length and internal ether group, but unlike MEG-OH, it can be employed to add anti-fouling properties to the surface while simultaneously having the capability to attach probes to biosensor surfaces.

Importantly, the acyl chloride group can participate with facility in a variety of reactions due to its sensitivity to nucleophilic attack. A monolayer comprised of this surface linker can be extended by reaction with an alcohol to form an ester [10], or an amine under basic conditions to form an amide [11,12]. In addition, the acyl chloride is resistant to reaction with the trichlorosilane group, preventing auto-cyclization of the molecule. The molecule has also been found to be stable when properly stored under nitrogen for several months.

We have previously used MEG-Cl in a biosensing context for linking gelsolin to a surface for use in detection of lysophosphatidic acid [13]. For this sensor multiple surface linkers were tested and it was found that MEG-Cl outperformed the other linkers for use in a sensor. However in that work the anti-fouling nature of MEG-Cl was not explored due to the nature of that biosensor. As anti-fouling is very important for many other types of biosensors, such as transduction biosensors mentioned above, the anti-fouling and linking properties of MEG-Cl on such a class of devices is herein explored.

**Scheme 1:**
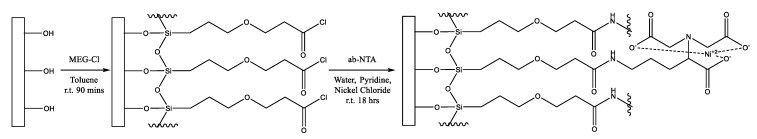
Surface modification with MEG-Cl followed by extension with ab-NTA to form a MEG-NTA surface coating**.**

In this work, we demonstrate the anti-fouling properties of this layer, as well as the usefulness of this linker by extending it with *N*_α_,*N*_α_-bis(carboxymethyl)-l-lysine to form MEG-NTA on the surface (Scheme 1). This was performed on the quartz substrate of an electromagnetic piezoelectric acoustic sensor (EMPAS) developed in our laboratory [14,15,16]. This device is particularly attractive for assessment of the extent of surface fouling from serum and other samples, such as milk, as shown in this work, which contain proteins.

## 2. Experimental

### 2.1. Materials

MEG-Cl and MEG-TFA were synthesized according to previously published methods [4,8]. Anhydrous Toluene, and *N*_α_,*N*_α_-bis(carboxymethyl)-l-lysine (ab-NTA) disodium salt monohydrate, were purchased from Sigma–Aldrich (St. Louis, MO, USA). Ethanol was obtained from Caledon Laboratory Chemicals (Georgetown, ON, Canada). All chemicals were used without further purification. Human serum was collected from apparently healthy donors at St. Michael’s Hospital (Toronto, ON, Canada). UHT milk was purchased from Walmart.

### 2.2. Cleaning and Surface Modification of Quartz Crystals

Quartz crystals (AT-cut, ⌀=13mm diameter, t=83 μm thickness, 20 MHz fundamental frequency) were purchased from Laptech Precision Inc. (Bowmanville, ON, Canada), were first sonicated in 20 mL of 1% SDS for 25 min, then thoroughly rinsed with hot tap water (x3) followed by distilled water (x3). Next, the crystals were individually soaked in 6 mL of Piranha solution (3:1 *v/v* mixture of 98% H_2_SO_4_ and 30% H_2_O_2_), pre-heated to 90°C using a water bath. After 30 min, the crystals were rinsed with distilled water (x3) followed by methanol (x3). The crystals were next sonicated in another portion of methanol for 2 min, and then placed in an oven maintained at 150 °C for drying. After 2 h, the crystals were further treated with plasma for 15 min, to increase the surface cleaning and hydroxylation and immediately transferred into a humidity chamber (70%–80% RH, room temperature) for overnight surface hydration.

Neat MEG-TFA or MEG-Cl (1 µL) was diluted with anhydrous toluene (1 mL) under inert (N_2_) and anhydrous (P_2_O_5_) atmosphere in a glovebox (Scheme 1). The solution was added to test tubes (pre-silanized with trichloro(octadecyl)silane) containing a cleaned quartz crystal. The vials were sealed, removed from the glovebox and placed on a spinning plate for 1.5 h. The crystals were then rinsed with anhydrous toluene and sonicated in deionized water for 5 min. The crystals were rinsed again with deionized water. MEG-TFA coated crystals were then submerged in 50% ethanol in distilled water, and rotated overnight to deprotect the surface layer leaving a MEG-OH coating. After this the crystals were rinsed with distilled water and dried.

For some MEG-Cl crystals a solution of ab-NTA and nickel(II) chloride (2 mg/mL ab-NTA and 2 mg/mL nickel(II) chloride in deionized water, 1 mL) was added to each test tube along with pyridine (0.5 mL) and placed on a spinning plate overnight (Scheme 1). These crystals, now modified with MEG-NTA, were rinsed with deionized water.

### 2.3. Contact Angle Goniometry (CAG)

Static contact angle measurements were performed for the clean and bare quartz discs, and each step of chemical surface modification. Surfaces were analyzed with the KSV CAM 101 contact angle goniometer (KSV Instruments Ltd., Helsinki, Finland) using type I water (18.20 MΩ cm) as the test liquid. Contact angle values were generated by the software provided with the instrument. Two discs were prepared for each step of the modification. Each side of each disc was tested twice.

### 2.4. EMPAS Measurements

The primary medium used in these experiments was 10 mM, pH 7.4 phosphate buffered saline (PBS, 10mM Na_2_HPO_4_, 154mM NaCl). Recombinant gelsolin solution was prepared at 0.4 mg/mL concentration as positive control. Albumin solution (0.4 mg/mL) was prepared by dissolving 0.4 mg of solid albumin into 1 mL of PBS buffer as negative control.

The experiments were run using an electromagnetic piezoelectric acoustic sensor (EMPAS), described previously [15]. After the standard set-up of EMPAS, MEG-OH and MEG-NTA SAM-coated quartz crystals were individually inserted into the flow through cell and PBS buffer was flown at a rate of 50 μL/min over the crystal.

EMPAS measurements were performed at the ultra-high frequencies of 1.06 GHz by measuring the crystal’s overtones and once the frequency signal stabilized, 50 μL of pooled human serum, milk, recombinant gelsolin or albumin samples were injected into the flow through system using a low-pressure chromatography valve. Once the sample completely passed over the surface, the uninterrupted PBS buffer flow rinsed the crystal surface of any loosely bound material. The frequency signal stabilized again, the experiment was stopped and the frequency shift (for non-specific adsorption) was calculated.

## 3. Results and Discussion

**Figure 3:**
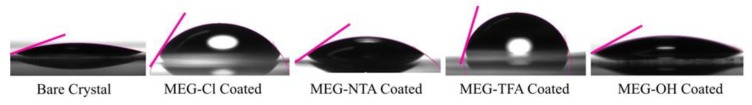
Images of water drops on quartz crystals at each modification step used to measure the contact angles and hydrophobicity of each.

XPS analysis of the surfaces has been previously studied [4,8] so measurement of contact angles for each step of quartz surface modification was considered sufficient to confirm that the substrates were successfully coated with the desired layer (Figure 3). Bare crystals showed a very high hydrophobicity, with contact angles of 17 ± 1 degrees on average. After modification with MEG-Cl the surface becomes more hydrophobic with the contact angle increasing to 58 ± 2 degrees on average. Extending MEG-Cl to form MEG-NTA results in more hydrophilic crystals with a reduced contact angle of 28 ± 1 degrees on average. This is expected due to the large number of hydrophilic carboxylic groups that are introduced to the surface with the addition of Ni-NTA.

The analogous system studied for comparison, MEG-OH, is first applied to the surface in its protected form, MEG-TFA, which renders the surface very hydrophobic displaying a contact angle of 78 ± 3 degrees on average. Following de-protection to produce a MEG-OH coated surface the contact angle decreases to 28 ± 1 degrees on average, which is expected due to the hydrophilic nature of MEG-OH.

The acoustic wave instrument used to test the level of surface fouling produced on quartz crystals by exposure to serum or milk samples typically operated at approximately 1065 MHz. The instrument also produced a background noise of 300–400 Hz, which was factored into the standard deviation of all averaged measurements. Unmodified quartz crystals were used as a baseline for fouling against serum or milk, and MEG-OH coated samples were used as the baseline for anti-fouling capability which we can compare MEG-Cl coated samples to.

**Figure 4:**
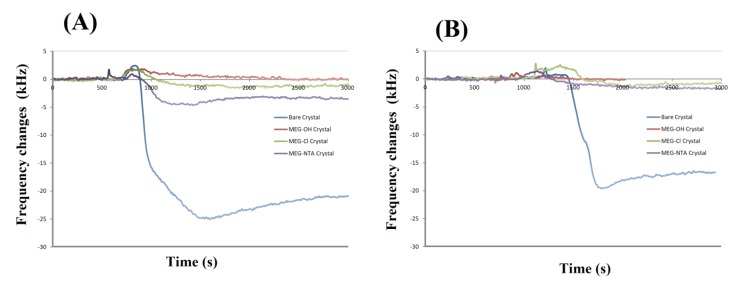
Examples of electromagnetic piezoelectric acoustic sensor (EMPAS) runs of bare and variously coated crystals exposed to serum (**A**) or milk (**B**).

Example experimental runs of frequency change versus time for bare and coated crystals are shown in Figure 4. Experimental runs such as these were used to prepare Table 1. From this it can be seen that MEG-OH, MEG-Cl and MEG-NTA coated crystals display excellent anti-fouling properties. Some experiments show a slight frequency increase after injection of serum or milk, which is a result of a slight pressure change in the system following injection. This effect quickly disappears once the pressure has returned to pre-injection levels.

Any fouling of the system was seen as a decrease in frequency from the established baseline as a result of protein mass adsorbing to the crystal surface. Bare crystals were used to determine the frequency change of a fully fouled surface, which modified surfaces could then be compared to. A smaller decrease in frequency from the baseline would be a result of a reduction in surface fouling.

**Figure 5:**
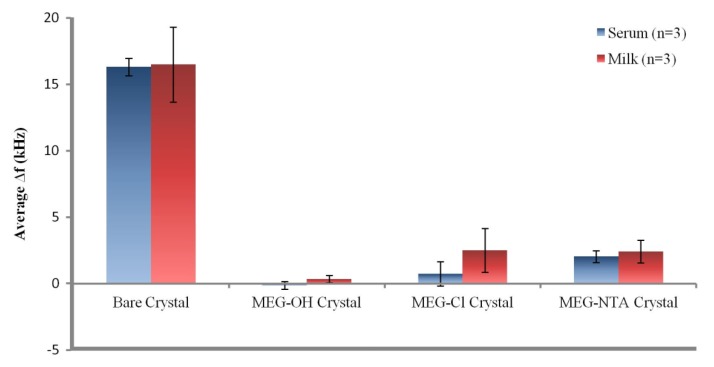
Visualization of the data from Table 1.

From the data in Table 1 it can be seen that the bare crystals showed the greatest level of fouling with an over 16 kHz drop in signal after exposure to either serum or milk. This suggests a large amount of non-specific adsorption from proteins and other biological molecules on the surfaces of the measured crystals. The data presented in Table 1 is visualized in Figure 5.

Coating crystals with MEG-OH before measuring them resulted in virtually no fouling being observed. The signals from each run nearly fully recovered to their baseline levels after exposure to either serum or milk, and washing with PBS buffer. Though a slight negative signal was observed for MEG-OH coated crystals exposed to serum, meaning the recovered signal was greater than the baseline signal, this measurement was within error of no change from baseline to recovery. This lack of frequency change from the baseline frequency suggests that there was virtually no adsorption of serum or milk species onto the crystal as a result of the MEG-OH coating. This baseline of almost complete prevention of biological fouling is in line with our previously published work [5], and compares favourably to other anti-fouling methods [3]. This reduction in fouling is what the results of MEG-Cl coated crystals will be compared to.

For MEG-Cl coated crystals a large amount of anti-fouling ability was observed, though not quite as good as MEG-OH. An average reduction in signal after serum fouling of 0.7 ± 0.9 kHz was observed, and 2.5 ± 1.7 kHz for milk exposed crystals, compared to the almost negligible change observed for MEG-OH. However, compared to the greater than 16 kHz change observed for non-coated crystals, this is a very large reduction in the level of fouling as a direct result of MEG-Cl surface coatings, representing an approximate 90% reduction in surface fouling which compares favourably to other anti-fouling methods [3]. This suggests strongly that MEG-Cl is able to prevent most serum and milk species from non-specifically adsorbing to the crystal surface, and is a capable anti-fouling layer.

The final fouling experiments involved MEG-Cl which has been extended with ab-NTA to form MEG-NTA on the surface which was tested against serum and milk in the EMPAS system. Crystals coated with MEG-NTA showed a small signal reduction after wash-off of 2.0 ± 0.4, and 2.4 ± 0.9 kHz following serum and milk exposure respectively. Although this is much greater fouling than was observed for MEG-OH, it is small compared to uncoated crystals suggesting MEG-NTA retains most of its anti-fouling ability even with the extended NTA group. This is very promising for the function of biosensors in biological samples which require a linker and probe system for their sensing capability. Such a linking system would allow for the binding of a desired probe to the sensing surface, while also preventing the majority of non-specific adsorption allowing for measurements to be carried out in biological samples with minimal background interference.

**Figure 6:**
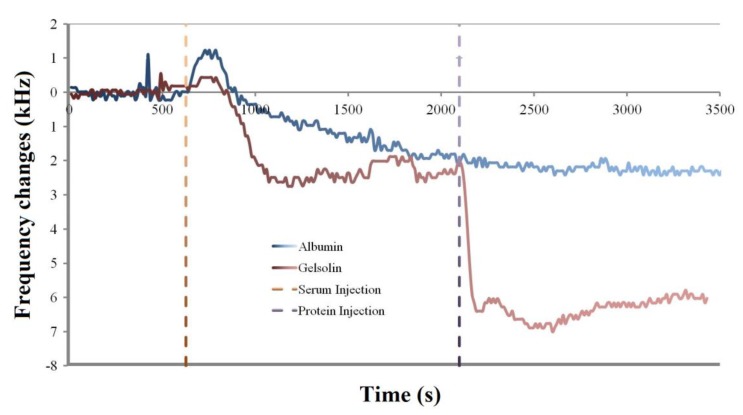
MEG-NTA coated quartz crystals first exposed to serum at 600 s, followed by injection of either albumin or gelsolin as measured by the EMPAS.

Another experiment was performed where either albumin or his tag-modified gelsolin protein was injected over an already fouled MEG-NTA quartz crystal to determine if the NTA would be able to selectively bind the gelsolin and function as a biosensing probe (Figure 6).

As can be seen in Figure 5 the expected ~2.5 kHz of fouling following serum injection over the crystals was observed. If the crystal was then exposed to albumin, which lacks a histidine tag for binding to Ni-NTA, there was no observed change in the frequency of the crystal (Figure 6 blue trace). The frequency continued to hover at ~2.5 kHz lower frequency than the baseline for the remainder of the experiment, suggesting that albumin was unable to bind to the already fouled crystal surface.

However following the injection of gelsolin after serum fouling the MEG-NTA crystal, the average frequency dropped to 6 kHz below the baseline frequency (Figure 6 red trace). This suggests that the histidine tagged gelsolin not only was specifically bound by the MEG-NTA probe group, but that this specific binding can occur even after the crystal has been fouled by biological fluids. This lends strong evidence to the usefulness of MEG-Cl for anti-fouling a biosensing surface as well as for binding specific target molecules to the surface for measurement, demonstrating its use in biosensing applications.

As mentioned in the introduction MEG-Cl has been used in conjunction with Ni-NTA to bind to a silica surface the lysophosphatidic acid (LPA) sensitive probe group of gelsolin and actin [13]. In that work a large increase in signal was observed for LPA when MEG-Cl was used as the surface linker versus a non-fouling surface linker in perfluorophenyl 12-(trichlorosilyl)dodecanoate. As well the biosensor was able to work well in whole serum, and not just in buffer solutions. This showed that in a practical biosensing application that MEG-Cl performs well as a surface linker, and combined with this work has great anti-fouling potential as well.

We have, additionally, developed a new biosensor for K^+^ to be employed for the detection of the cation in real-time in the vagus nerve [17]. This involves tandem MEG-Cl anti-fouling chemistry with attachment of a newly synthesized crown-ether based probe for selective binding of the cation. Preliminary experiments reveal that the biosensor operates successfully for K^+^ detection in cerebrospinal fluid.

This work has shown that the strong anti-fouling and surface linking capabilities of MEG-Cl make it a highly promising surface linker in the context of biosensor technology, as it can allow a biosensor to operate in biological fluids such as serum, cerebrospinal fluid or milk, MEG-Cl will allow researchers to bring their biosensors out of the lab and buffers and into real world clinical applications.

## 4. Conclusions

A surface monolayer produced from 3-(3-(trichlorosilylpropyloxy)propanoyl chloride has been successfully shown to significantly reduce fouling caused by biological fluids in a flow-through acoustic wave biosensor. It has also been found that the layer is still able to prevent a large amount of surface fouling even after it has been functionalized with a protein capturing group ab-NTA. In addition, the NTA group is able to selectively capture his-tagged proteins even in the presence of serum. Since the trichlorosilyl group of the product may react with hydroxyl groups in the presence of water to form a surface monolayer, this surface chemistry may be extended to any hydroxylated surface. Further applications of this chemistry will be explored in the biosensing context, by using this layer to bind a biosensing probe to the acoustic sensing surface and detecting a desired molecule in biological fluids.

## Figures and Tables

**Table 1:** Results of fouling experiments where the baseline is recorded, and fouling measured by looking at the resulting frequency after sample exposure to biological fluid and washing with PBS. Averages and overall standard deviation are shown for n experimental runs.

Crystal Modification	Fluid Exposed	Avg. Baseline (kHz)	Avg. Recovery (kHz)	Avg. Δf ± St. Dev. (kHz)
**Bare crystal**	Serum (n=3)	1,065,256	1,065,240	**16.3 ± 0.65**
	Milk (n=3)	1,064,806	1,064,790	**16.5 ± 2.81**
**MEG-OH crystal**	Serum (n=3)	1,065,138	1,065,138	**−0.10 ± 0.30**
	Milk (n=3)	1,065,226	1,065,225	**0.35 ± 0.27**
**MEG-Cl crystal**	Serum (n=3)	1,065,695	1,065,694	**0.74 ± 0.92**
	Milk (n=3)	1,065,374	1,065,371	**2.52 ± 1.65**
**MEG-NTA crystal**	Serum (n=3)	1,065,141	1,065,225	**2.04 ± 0.44**
	Milk (n=3)	1,065,040	1,065,037	**2.42 ± 0.86**
